# NudCL2 regulates cell migration by stabilizing both myosin-9 and LIS1 with Hsp90

**DOI:** 10.1038/s41419-020-02739-9

**Published:** 2020-07-14

**Authors:** Wenwen Chen, Wei Wang, Xiaoxia Sun, Shanshan Xie, Xiaoyang Xu, Min Liu, Chunxia Yang, Min Li, Wen Zhang, Wei Liu, Liangjing Wang, Tianhua Zhou, Yuehong Yang

**Affiliations:** 1 Department of Cell Biology, and Institute of Gastroenterology of the Second Affiliated Hospital, Zhejiang University School of Medicine, Hangzhou Zhejiang, 310058 China; 2 Shanghai Key Laboratory of Psychotic Disorders, Shanghai Mental Health Center, Shanghai Jiao Tong University School of Medicine, Shanghai, 201108 China; 3 The Cancer Center of the Second Affiliated Hospital, Zhejiang University School of Medicine, Hangzhou Zhejiang, 310009 China; 4 Collaborative Innovation Center for Diagnosis and Treatment of Infectious Diseases, Hangzhou Zhejiang, 310003 China; 5 Department of Molecular Genetics, University of Toronto, Toronto, Canada

**Keywords:** Cell migration, Cytoskeleton, RNAi

## Abstract

Cell migration plays pivotal roles in many biological processes; however, its underlying mechanism remains unclear. Here, we find that NudC-like protein 2 (NudCL2), a cochaperone of heat shock protein 90 (Hsp90), modulates cell migration by stabilizing both myosin-9 and lissencephaly protein 1 (LIS1). Either knockdown or knockout of NudCL2 significantly increases single-cell migration, but has no significant effect on collective cell migration. Immunoprecipitation–mass spectrometry and western blotting analyses reveal that NudCL2 binds to myosin-9 in mammalian cells. Depletion of NudCL2 not only decreases myosin-9 protein levels, but also results in actin disorganization. Ectopic expression of myosin-9 efficiently reverses defects in actin disorganization and single-cell migration in cells depleted of NudCL2. Interestingly, knockdown of myosin-9 increases both single and collective cell migration. Depletion of LIS1, a NudCL2 client protein, suppresses both single and collective cell migration, which exhibits the opposite effect compared with myosin-9 depletion. Co-depletion of myosin-9 and LIS1 promotes single-cell migration, resembling the phenotype caused by NudCL2 depletion. Furthermore, inhibition of Hsp90 ATPase activity also reduces the Hsp90-interacting protein myosin-9 stability and increases single-cell migration. Forced expression of Hsp90 efficiently reverses myosin-9 protein instability and the defects induced by NudCL2 depletion, but not vice versa. Taken together, these data suggest that NudCL2 plays an important role in the precise regulation of cell migration by stabilizing both myosin-9 and LIS1 via Hsp90 pathway.

## Introduction

Cell migration plays a pivotal role in many fundamental biological processes, including embryonic development, tissue homeostasis, immune surveillance, and wound healing^1–3^. In general, there exist two basic types of cell migration in vivo and in vitro: single-cell migration (the movement of cells independent of each other) and collective cell migration (cell movement in a mass in which cell–cell contact is maintained)^4,5^. Defects in cell migration are involved in a range of human diseases, such as autoimmune syndromes, mental retardation, cancer, and so on^3,6^. However, the precision mechanism of cell migration regulation remains unclear.

Accumulating studies indicate that the dynamics of actin cytoskeleton regulates cell migration spatially and temporally^7^. Myosins are a large family of actin-based molecular motors that bind actin filaments to generate force and movement^8,9^. Nonmuscle myosin IIA (NM IIA) belongs to the myosin II subfamily and is a hexamer composed of two heavy chains (named myosin-9, also known as NMMHC-IIA) and two pairs of essential and regulatory light chains (MELCs and MRLCs)^9–14^. NM IIA is found to polymerize into bipolar minifilaments that interact with actin filaments via their ATPase head. NM IIA minifilaments slide actin filaments in an anti-parallel manner by using the energy derived from ATP hydrolysis^10^. The assembly and disassembly of NM IIA is mainly regulated by myosin-9 phosphorylation via protein kinase C (PKC) and casein kinase II (CK II)^11^. However, the regulation of myosin-9 protein stability is largely unknown.

NudC-like protein 2 (NudCL2), a homolog of mammalian nuclear distribution gene C (NudC, a key regulator of the LIS1/dynein pathway), was originally cloned and identified as a cochaperone of heat shock protein 90 (Hsp90) by our group^15–18^. Our works have shown that NudCL2 stabilizes LIS1 (lissencephaly protein 1) by enhancing the interaction between LIS1 and Hsp90^18^. Recently, we find that NudCL2 is also involved in the regulation of sister chromatid cohesion and centriole duplication by stabilizing cohesin subunits and E3 ligase HECT domain and RCC1-like domain-containing protein 2 (HERC2), respectively^15,19^. However, little is known about the role of NudCL2 in cell migration.

In this study, we provide evidence that NudCL2 is required for cell migration regulation. Our data show that downregulation of NudCL2 promotes single-cell migration, but has no significant effect on collective cell migration. NudCL2 stabilizes both myosin-9 and LIS1 with Hsp90. Co-depletion of myosin-9 and LIS1 increases single-cell migration, resembling the phenotypes in NudCL2-depleted cells. Thus, we propose that NudCL2 plays an important role in the precise regulation of cell migration by modulating the stability of both myosin-9 and LIS1 with Hsp90, providing a hitherto unrecognized mechanism crucial for cell migration regulation.

## Results

### NudCL2 is required for cell migration

**Fig. 1:**
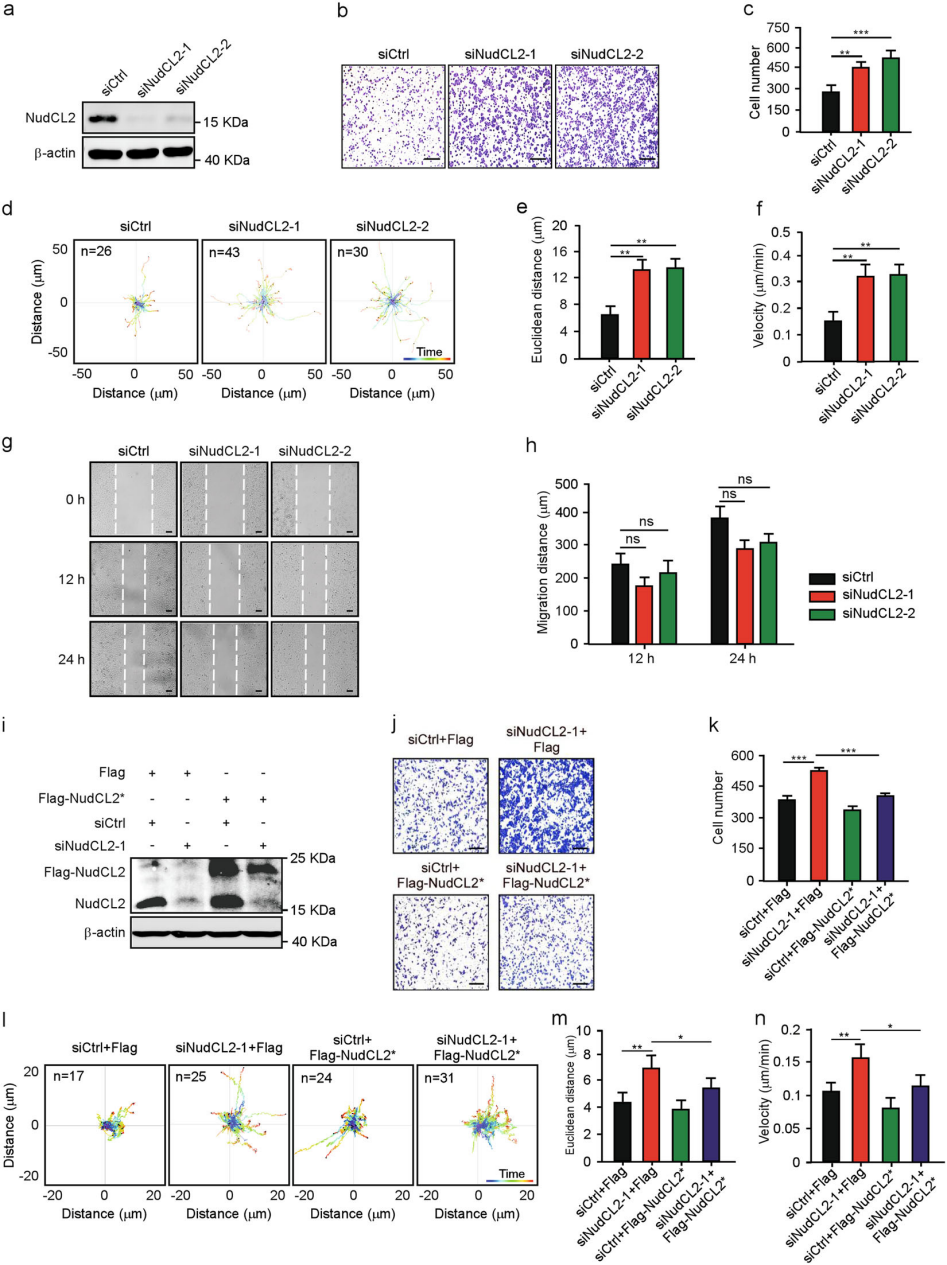
NudCL2 is required for single-cell migration in vitro. **a** A549 cells transfected with siRNAs targeting different *NudCL2* mRNA regions (siNudCL2-1 and siNudCL2-2) were subjected to western blotting analysis with anti-NudCL2 antibody. β-actin was used as a loading control. **b**, **c** Transwell migration assays revealed the cell motility of control and NudCL2-depleted cells. Scale bar, 200 μm. Cells that migrated to the undersides of the filters were counted. **d**–**f** The migration tracks of individual cells transfected with the indicated siRNAs were traced by Imaris 9.1.2 software. Representative single-cell migration paths are shown. Euclidean distance and migration velocity were calculated. **g**, **h** The wound healing assays showed collective cell migration at different time points. Dashed lines indicate the wound edges. Scale bar, 200 μm. The distance of the wound was measured by ImageJ software. **i**–**k** Cells transfected with the indicated siRNAs and Flag-NudCL2* (siRNA-resistant NudCL2) or Flag were subjected to the following analyses. Western blotting analysis showed the expression of NudCL2 and Flag-NudCL2. β-actin was used as a loading control. Transwell migration assays revealed cell motility. Scale bar, 200 μm. Cells that migrated to the undersides of the filters were counted. **l**–**n** Cells transfected with the indicated siRNAs and vectors for 72 h were subjected to a migration experiment. The migration paths of the individual cells were analyzed with Imaris 9.1.2 software. Representative single-cell migration tracks are shown. Euclidean distance and migration velocity were measured. Quantitative data from at least three independent experiments are shown as the mean ± SD. *n*, sample size. *P < 0.05; **P < 0.01; ***P < 0.001; ns, not significant (*P* > 0.05). Student’s *t*-test.

To explore the role of NudCL2 in cell migration, we employed small interfering RNAs (siRNAs) to deplete NudCL2. We used two siRNA oligos targeting two different regions of *NudCL2* mRNA (siNudCL2-1 and siNudCL2-2) and found that the protein levels of NudCL2 was substantially reduced 72 h post-transfection (Fig. 1a). Transwell migration assays showed that depletion of NudCL2 increased single-cell migration (Fig. 1b, c). Tracing the migratory path of live cells by time-lapse microscopy revealed that knockdown of NudCL2 increased the speed of single-cell motility (Fig. 1d–f). Interestingly, wound healing assay showed that downregulation of NudCL2 had no significant effect on collective cell migration (Fig. 1g, h). Furthermore, exogenic expression of siRNA-resistant NudCL2 was able to reverse the defects in single-cell migration induced by NudCL2 depletion (Fig. 1i–n). The similar phenomenon was also found in HeLa and HEK-293 cells (Supplementary Figs. 1 and 2). To further confirm the role of NudCL2 in cell movement, we generated a *NudCL2* knockout (*NudCL2* KO) A549 cell line using CRISPR/Cas9-mediated gene editing technique. The data showed that deletion of NudCL2 also significantly increased single-cell migration, but not collective cell migration (Supplementary Fig. 3). Taken together, our results strongly indicate that NudCL2 is essential for single-cell migration in mammalian cells.

### NudCL2 binds to and stabilizes myosin-9

**Fig. 2:**
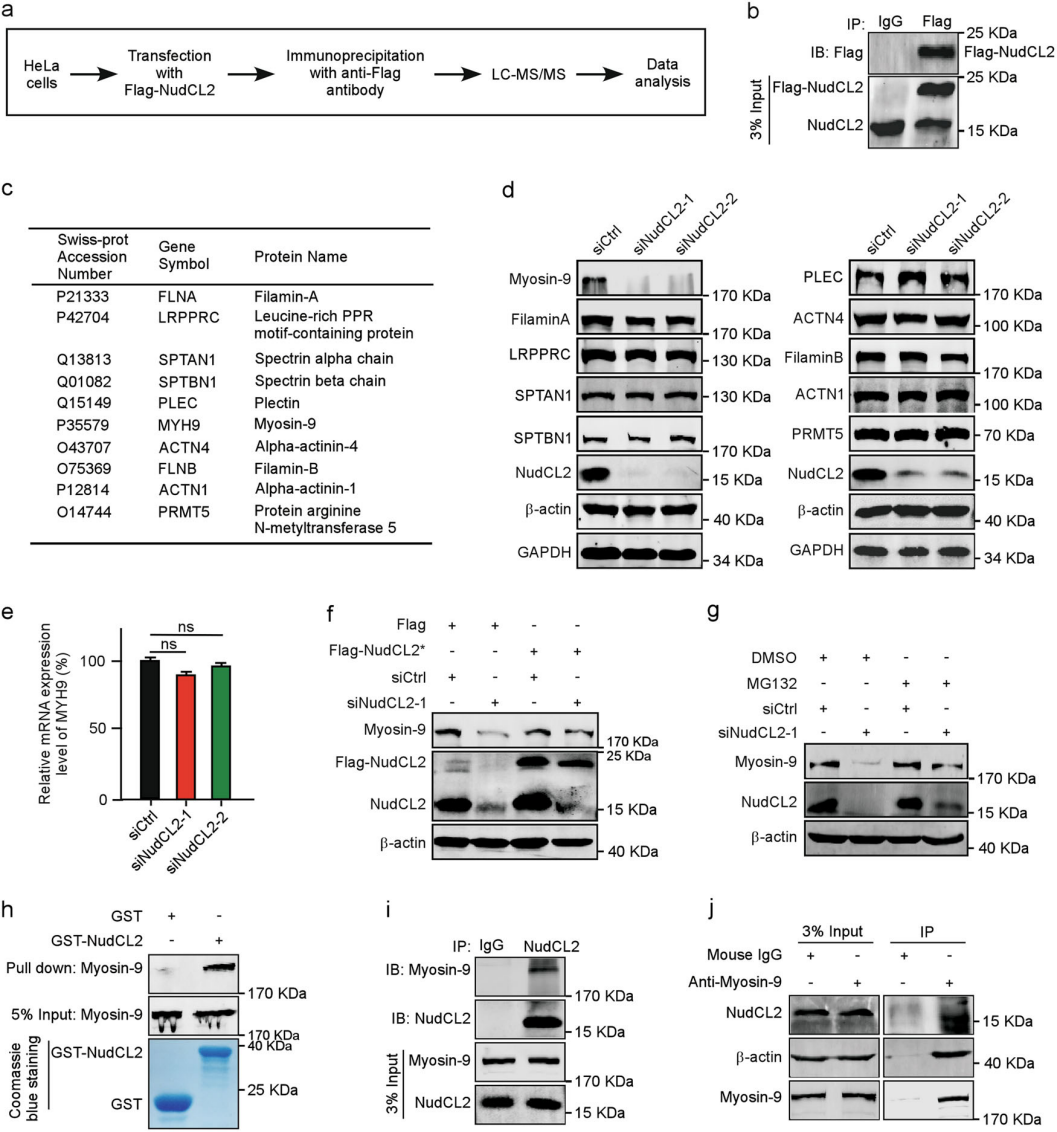
NudCL2 binds to and stabilizes myosin-9. **a** Schematic representation of the IP-Mass approach. **b** HeLa cells transfected with either Flag-NudCL2 or Flag were subjected to immunoprecipitation analysis with anti-Flag antibody-coupled beads. **c** The top 10 proteins ranked by relative abundance from IP-Mass analysis are shown. **d** A549 cells transfected with control or NudCL2 siRNA were subjected to western blotting analysis with the indicated antibodies. GAPDH was used as a loading control. **e** Quantitative RT-PCR analysis of *MYH9* mRNA in control and NudCL2-depleted cells. GAPDH was used as an internal control. **f** A549 cells transfected with the indicated siRNAs and vectors were subjected to western blotting analysis with anti-NudCL2 and anti-Myosin-9 antibodies. β-actin was used as a loading control. **g** Cells were treated with 10 µM MG132 or DMSO for 2 h. Cell lysates were used for western blotting analysis with anti-Myosin-9 and anti-NudCL2 antibodies. β-actin was used as a loading control. **h** Purified GST or GST-NudCL2 protein was incubated with A549 cell lysates and subjected to immunoblotting with anti-Myosin-9 antibody. Five percent of the total input is shown. GST and GST-NudCL2 input was stained with Coomassie brilliant blue. **i**, **j** A549 cells were harvested and lysed. Immunoprecipitation analyses were carried out using the indicated antibodies. Three percent of the total input is shown. Quantitative data are expressed as the mean ± SD (at least three independent experiments). ns, no significance (*P* > 0.05). Student’s *t*-test.

To explore the underlying mechanism of NudCL2 in cell migration regulation, we employed immunoprecipitation (IP) assay coupled with liquid chromatography/tandem mass spectrometry (LC-MS/MS)-based interactome approach to screen the potential NudCL2-interacting proteins (Fig. 2a). HeLa cells were transfected with Flag-NudCL2 vector and subjected to IP analysis. Western blotting confirmed that Flag-NudCL2 was efficiently immunoprecipitated with anti-Flag antibody-coupled beads (Fig. 2b). Further mass spectrometry analysis revealed that approximate 343 proteins were possibly associated with Flag-NudCL2 (Supplementary Table 1, ranked based on relative abundance). Among them, most of top 10 proteins were actin-related proteins, including filamin-A, spectrin alpha chain, spectrin beta chain, plectin, myosin-9, alpha-actinin-4, filamin-B, and alpha-actinin-1 (Fig. 2c). Since our previous data demonstrated that NudCL2 functions as an Hsp90 cochaperone to regulate client proteins’ stability^15,18,19^, we examined the protein levels of these proteins in NudCL2-depleted cells. Western blotting showed that only myosin-9 protein levels were substantially decreased in NudCL2-depleted A549 cells (Fig. 2d), RT-PCR showed that myosin-9 mRNA level was not changed (Fig. 2e). Further data showed that the downregulation of myosin-9 protein in NudCL2-depleted cells could be reversed by ectopic expression of siRNA-resistant NudCL2 (Fig. 2f). Moreover, we treated NudCL2-depleted cells with the proteasome inhibitor MG132 and found that MG132 inhibited the degradation of myosin-9 (Fig. 2g), implying that the ubiquitin-proteasome pathway maybe involved in myosin-9 degradation. In addition, GST pull-down assays revealed that NudCL2 was able to interact with myosin-9 in vitro (Fig. 2h). In addition, IP assays showed that NudCL2 associated with myosin-9 in vivo (Fig. 2i, j). Taken together, these data suggest that NudCL2 is required for the stabilization of the NudCL2-binding protein myosin-9.

### Depletion of NudCL2 impairs actin dynamics

**Fig. 3:**
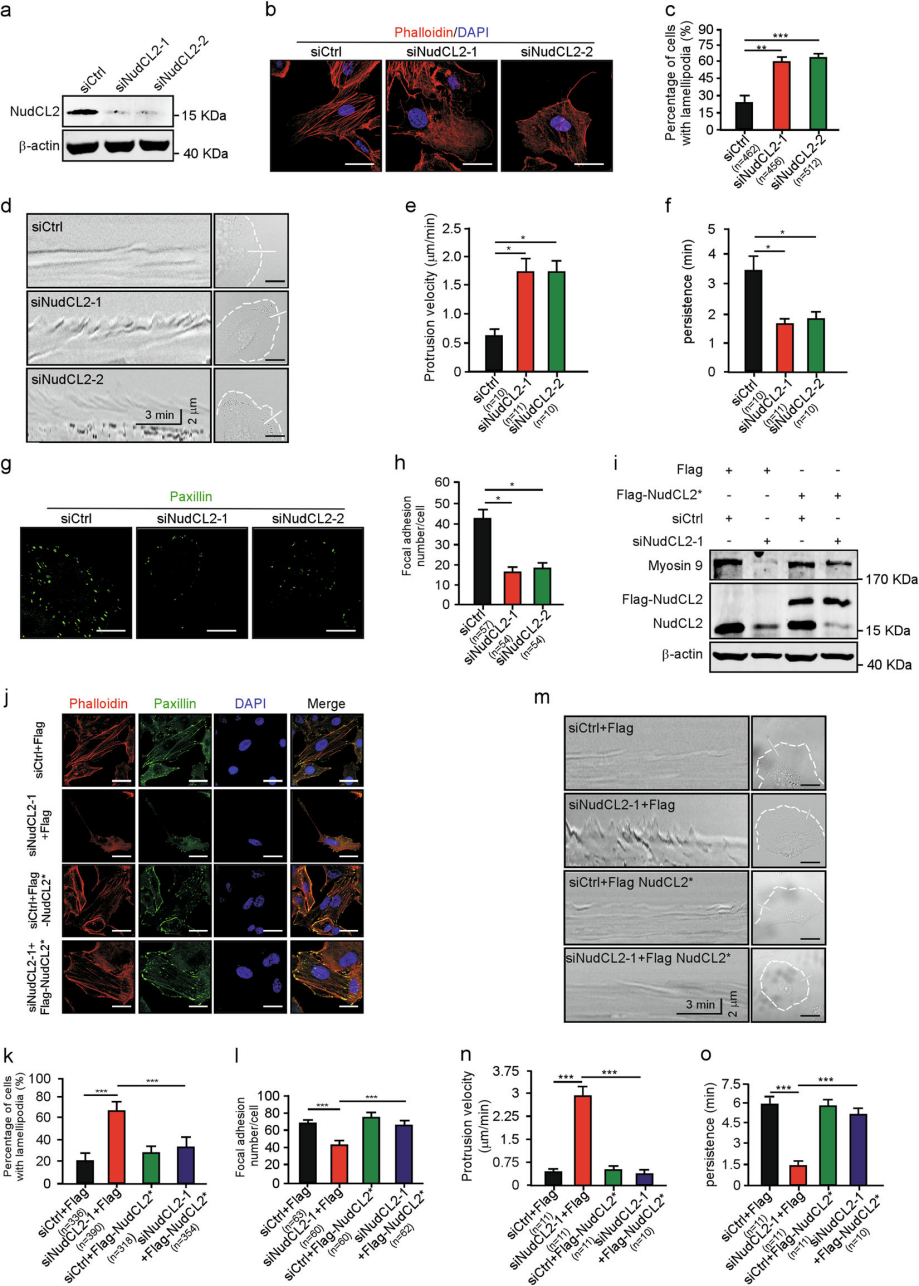
Depletion of NudCL2 impairs actin dynamics. A549 cells transfected with siRNAs and vectors were subjected to the following analyses: **a** Western blotting analysis of the expression of NudCL2. β-actin was used as a loading control. **b** Cells were fixed and stained with phalloidin (red). DNA was visualized with DAPI (blue). Scale bar, 20 μm. **c** The percentage of cells with lamellipodia in (**b**) was calculated. More than 100 cells were counted in each experiment. **d** A sequence of phase-contrast time-lapse images of the cells were obtained with a LSM880 confocal microscope using a ×63 objective. Kymographs were produced and analyzed using MetaMorph software. The minimum intensity projection of a 250-frame movie (3 s per frame) is presented on the left. Pixel intensities along a one-pixel-wide line (white) were used to generate the kymograph presented on the right. Cells are outlined with dashed lines. Scale bar, 20 μm. **e**, **f** The velocity and persistence of lamellipodia protrusions in (**d**) are shown. **g** Cells were fixed and subjected to immunofluorescence staining with anti-paxillin (green) antibody. Scale bar, 20 μm. **h** Focal adhesions between cells in (**g**) were counted. About 20 cells were counted in each experiment. **i** Cells transfected with the indicated siRNA and vectors were subjected to western blotting analysis using the antibodies as shown. β-actin was used as a loading control. **j**–**l** Cells were fixed and stained with phalloidin (red) and anti-paxillin (green) antibody. DNA was visualized with DAPI (blue). Scale bar, 20 μm. The percentage of cells with lamellipodia and number of cellular focal adhesions were plotted respectively. More than 100 cells were counted in each experiment. **m**–**o** A sequence of phase-contrast time-lapse images of cells were obtained with a LSM880 confocal microscope using a ×63 objective. Kymographs were produced and analyzed using MetaMorph software. The minimum intensity projection of a 250-frame movie (3 s per frame) is presented on the left. Pixel intensities along a one-pixel-wide line (white) were used to generate the kymograph presented on the right. Cells are outlined with dashed lines. Scale bar, 20 μm. The velocity and persistence of lamellipodia protrusions are calculated. Quantitative data derived from at least three independent experiments are shown as the mean ± SD. *n*, sample size. *P < 0.05; **P < 0.01; ***P < 0.001. Student’s *t*-test.

Given that myosin-9 regulates cell migration mainly by regulating actin organization and is stabilized by NudCL2 (Fig. 2)^8,20^, we investigate whether depletion of NudCL2 affects the structure and function of the actin cytoskeleton. Immunostaining assays using fluorescent phalloidin-labeled F-actin showed that depletion of NudCL2 led to a dramatic decrease in actin stress fiber formation and increase in lamellipodia formation at the cell leading edge (Fig. 3a–c). Kymographs of lamellipodial protrusion showed that downregulation of NudCL2 caused a significant increase in protrusion velocity with a concomitant decrease in protrusion persistence (Fig. 3d–f). Furthermore, depletion of NudCL2 caused obvious decrease in the number of focal adhesions by using paxillin staining (Fig. 3g, h). Rescue experiments revealed that these phenotypes induced by NudCL2 depletion were significantly reversed by exogenic expression of siRNA-resistant NudCL2 (Fig. 3i–o). Collectively, these data imply that NudCL2 may act as an important regulator of actin cytoskeleton organization and function.

### Ectopic expression of myosin-9 reverses the phenotypes induced by NudCL2 depletion

**Fig. 4:**
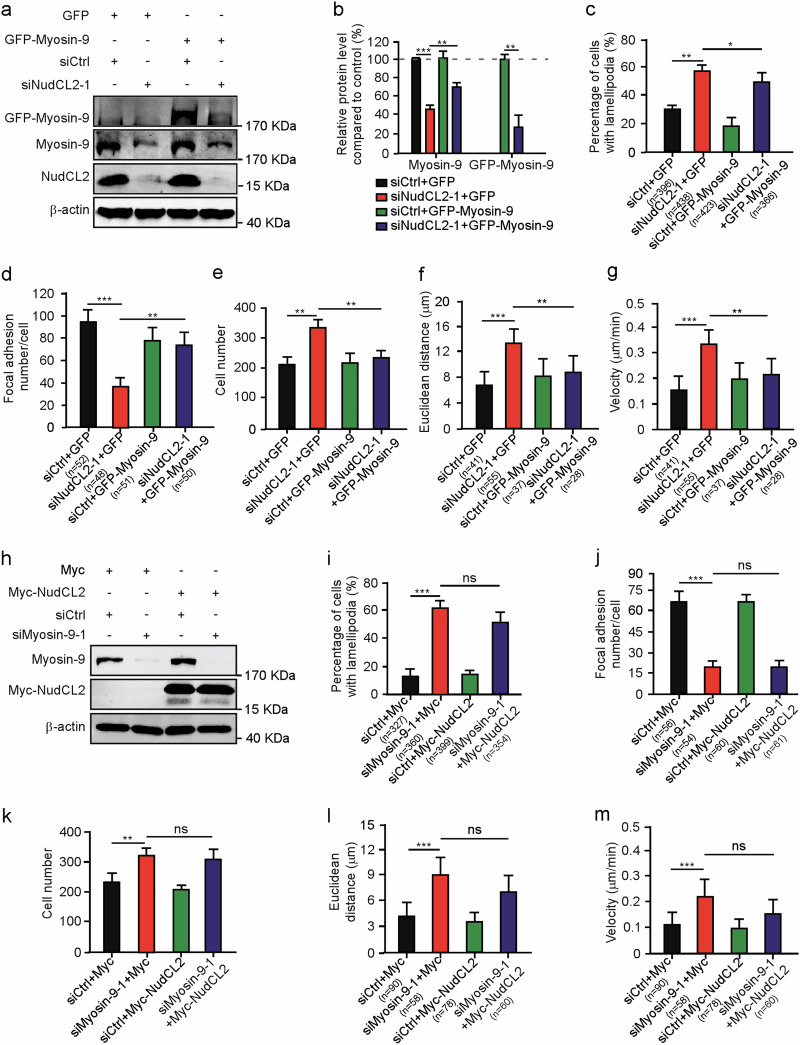
Exogenous expression of myosin-9 reverses the defects caused by NudCL2 depletion. A549 cells transfected with siRNAs and vectors were subjected to the following analyses: **a** Western blotting analysis of the expression of NudCL2, myosin-9, and GFP-myosin-9. β-actin was used as a loading control. **b** ImageJ software was used to quantify protein levels in (**a**). The relative amounts of myosin-9 and GFP-myosin-9 compared with the control were calculated and shown. **c**, **d** Cells were fixed and stained with phalloidin, anti-paxillin antibody and DAPI. Cells with lamellipodia were counted, and the number of cellular focal adhesions was plotted. **e** Transwell migration assays revealed cell motility. Cells that migrated to the undersides of the filters were counted. **f**, **g** The migration tracks of individual cells were traced by Imaris 9.1.2 software. Euclidean distance and migration velocity were measured. **h** A549 cells transfected with the indicated siRNAs and vectors were subjected to the following analyses: western blotting analysis of the expression of myosin-9 and Myc-NudCL2. β-actin was used as a loading control. **i**, **j** Cells were fixed and stained with phalloidin, anti-paxillin antibody, and DAPI. Cells with lamellipodia were counted, and the number of cellular focal adhesions was plotted. **k** Transwell migration assays revealed cell motility. Cells that migrated to the undersides of the filters were counted. **l**, **m** The migration tracks of individual cells were traced by Imaris 9.1.2 software. Euclidean distance and migration velocity were measured. Quantitative data derived from at least three independent experiments are shown as the mean ± SD. More than 100 cells were counted in each experiment. *n*, sample size. *P < 0.05; **P < 0.01; ***P < 0.001; ns, not significant (*P* > 0.05). Student’s *t*-test.

Since depletion of NudCL2 destabilizes myosin-9 and affects actin organization and cell migration, we test whether myosin-9 is involved in NudCL2-mediated cell migration. Our immunostaining, transwell migration assays and live cell migration assays displayed that exogenous expression of myosin-9 significantly reversed the defects in actin organization, focal adhesion formation, and cell migration induced by NudCL2 depletion (Fig. 4a–g), but not vice versa (Fig. 4h–m). Thus, these data suggest that myosin-9 may be a downstream target protein of NudCL2 in cell migration regulation.

### Co-depletion of myosin-9 and LIS1 phenocopies NudCL2 downregulation

**Fig. 5:**
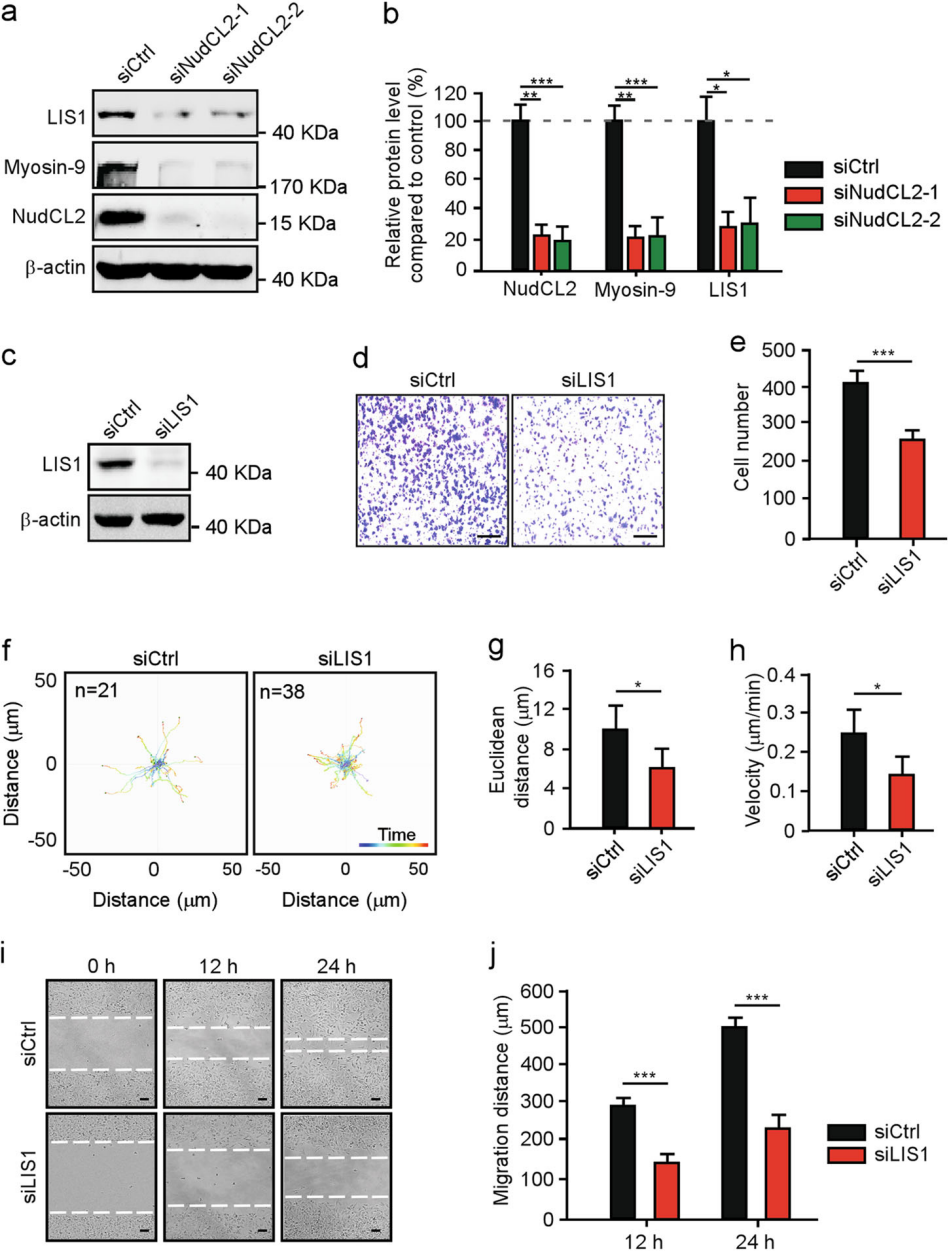
Depletion of LIS1 inhibits cell migration. A549 cells transfected with siRNAs were subjected to the following analyses: **a** Western blotting analysis of the expression of NudCL2, myosin-9, and LIS1. β-actin was used as a loading control. **b** ImageJ software was used to quantify protein levels in (**a**). The relative amounts of myosin-9 and LIS1 compared with the control were calculated and shown. **c** Western blotting analysis of the expression of LIS1. **d**, **e** Transwell migration assays revealed the cell motility of control and LIS1-depleted cells. Scale bar, 200 μm. Cells that migrated to the undersides of the filters were counted. **f**–**h** The migration tracks of individual cells were traced by Imaris 9.1.2 software. Representative single-cell migration paths are shown. Euclidean distance and migration velocity were measured. **i**, **j** Wound healing assays showed collective cell migration at different time points. Dashed lines indicate wound edges. Scale bar, 200 μm. The distance of the wound was measured by ImageJ software. Quantitative data derived from at least three independent experiments are shown as the mean ± SD. *n*, sample size. *P < 0.05; **P < 0.01; ***P < 0.001. Student’s *t*-test.

**Fig. 6:**
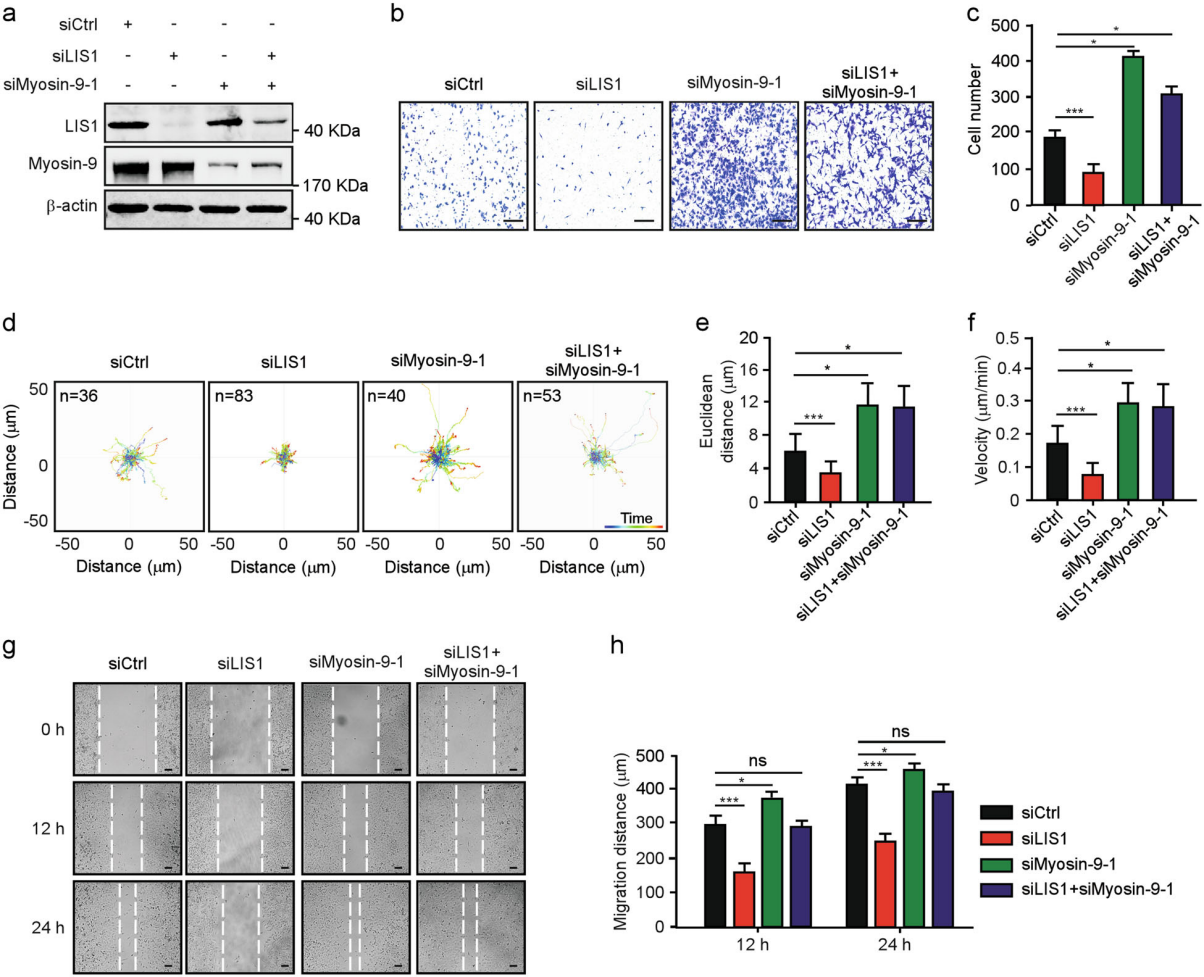
Co-depletion of myosin-9 and LIS1 promotes single-cell migration. A549 cells transfected with siRNAs were subjected to the following analyses: **a** Western blotting analysis of the expression of LIS1 and myosin-9. β-actin was used as a loading control. **b**, **c** Transwell migration assays revealed cell motility. Scale bar, 200 μm. Cells that migrated to the undersides of the filters were counted. **d**–**f** The migration tracks of individual cells were traced by Imaris 9.1.2 software. Representative single-cell migration paths are shown. Euclidean distance and migration velocity were measured. **g**, **h** Wound healing assays showed collective cell migration at different time points. Dashed lines indicate wound edges. Scale bar, 200 μm. The distance of the wound was measured by ImageJ software. Quantitative data derived from at least three independent experiments are shown as the mean ± SD. *n*, sample size. *P < 0.05; ***P < 0.001; ns, not significant (*P* > 0.05). Student’s *t*-test.

Previous reports showed that myosin-9 depletion promotes both single and collective cell migration in A549 cells^21,22^, which was consistent with our data (Supplementary Fig. 4). Given that depletion of NudCL2 only significantly affected single-cell migration (Fig. 1), one reasonable hypothesis is that there exists other protein, which plays a role in the regulation of NudCl2-mediated single-cell migration. Our previous works demonstrated that NudCL2 is able to stabilize LIS1, a key regulator of cell migration^18,23,24^; therefore, we carefully examined the protein level of LIS1 in cells depleted of NudCL2. The data revealed that knockdown of NudCL2 substantially caused LIS1 downregulation in A549 cells (Fig. 5a, b). Further results displayed that LIS1 depletion suppressed both single and collective cell migration, exhibiting the opposite effect compared with those of myosin-9 knockdown (Fig. 5c–j). Interestingly, co-depletion of myosin-9 and LIS1 only increased single-cell migration, but had no significant effect on collective cell migration, resembling the phenotypes caused by NudCL2 downregulation (Fig. 6). Taken together, these results strongly suggest that NudCL2 plays an important role in the precise regulation of cell migration by stabilizing both myosin-9 and LIS1.

### Hsp90 participates in the regulation of myosin-9 by NudCL2

**Fig. 7:**
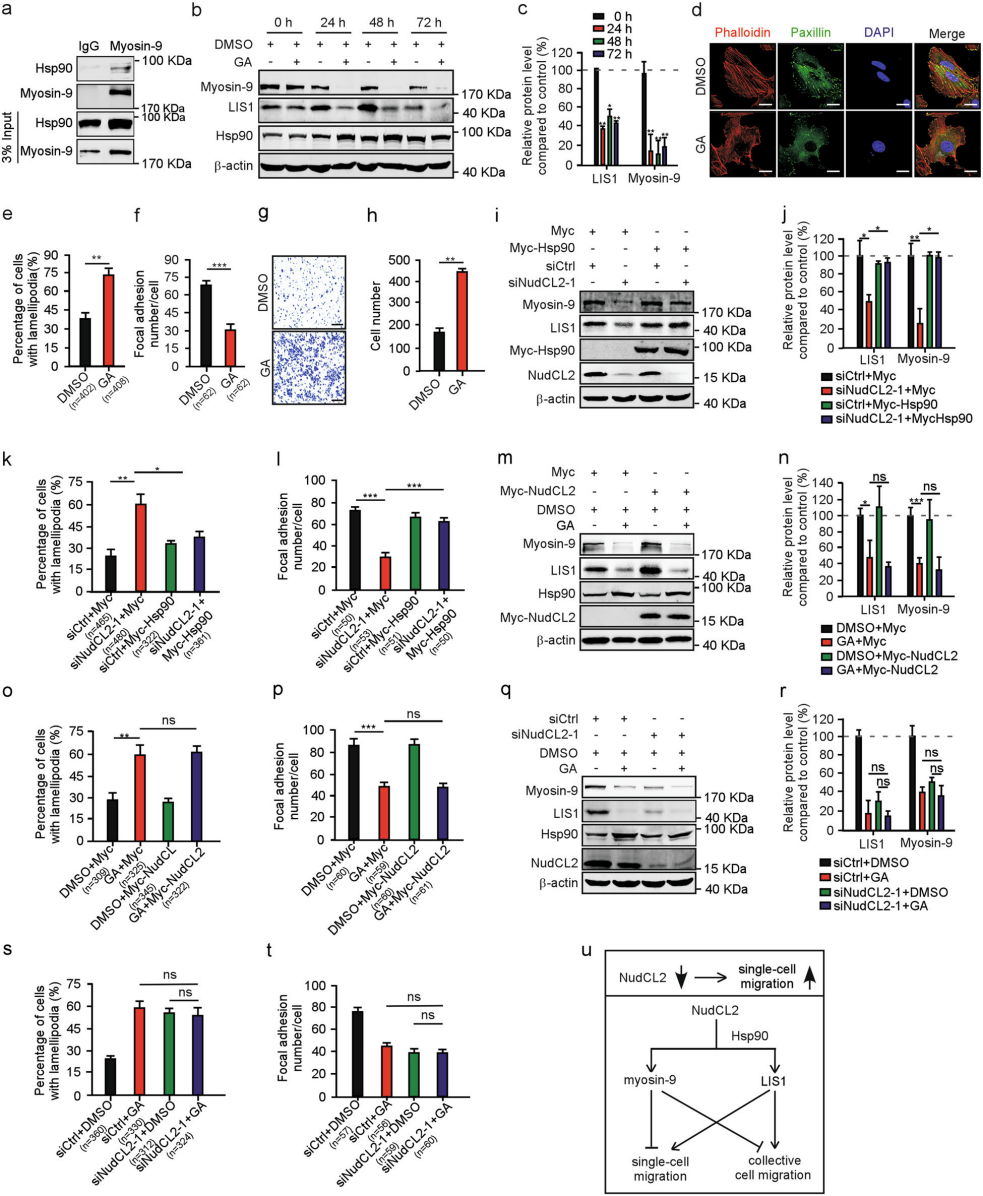
Hsp90 is involved in NudCL2-meidated myosin-9 stability and cell migration. A549 cells transfected with or without the indicated siRNAs and vectors were treated with 3.78 µM geldanamycin (GA) or DMSO for 48 h and subjected to the following analyses: **a** A549 cells were harvested and lysed. Immunoprecipitation analyses were carried out using the indicated antibodies. Three percent of the total input is shown. **b** Western blotting analysis of the expression of myosin-9, LIS1, and Hsp90. β-actin was used as a loading control. **c** Relative protein levels compared with the control at the same time point of GA treatment in (**b**) were measured using ImageJ software and shown. **d**–**f** Cells were fixed and stained with phalloidin (red) and anti-paxillin (green) antibody. DNA was visualized with DAPI (blue). Scale bar, 20 μm. Cells with lamellipodia were counted, and the number of cellular focal adhesions was plotted. **g**, **h** Transwell migration assays revealed cell motility. Scale bar, 200 μm. Cells that migrated to the undersides of the filters were counted. **i** Western blotting analysis of the expression of myosin-9, LIS1, Myc-Hsp90, and NudCL2. β-actin was used as a loading control. **j** ImageJ software was used to quantify protein levels in (**i**). The relative amounts of myosin-9 and LIS1 compared with the control were calculated and shown. **k**, **l** Cells were stained as described in (**d**). Cells with lamellipodia were counted, and the number of cellular focal adhesions was plotted. **m** Western blotting analysis of the expression of myosin-9, LIS1, Hsp90, and Myc-NudCL2. β-actin was used as a loading control. **n** ImageJ software was used to quantify protein levels in (**m**). The relative amounts of myosin-9 and LIS1 compared with the control were calculated and shown. **o**, **p** Cells were stained as described in (**d**). Cells with lamellipodia were counted, and the number of cellular focal adhesions was plotted. **q** A549 cells transfected with the indicated siRNAs were treated with GA and subjected to western blotting analysis of the expression of myosin-9, LIS1, Hsp90, and NudCL2. β-actin was used as a loading control. **r** ImageJ software was used to quantify protein levels in (**q**). The relative amounts of myosin-9 and LIS1 compared with the control were calculated and shown. **s**, **t** Cells were stained as described in (**d**). Cells with lamellipodia were counted, and the number of cellular focal adhesions was plotted. Quantitative data derived from at least three independent experiments are shown as the mean ± SD. More than 100 cells were counted in each experiment. *n*, sample size. *P < 0.05; **P < 0.01; ***P < 0.001; ns, not significant (*P* > 0.05). Student’s *t*-test. **u** Working model for the role of NudCL2 in cell migration. NudCL2 stabilizes myosin-9 and LIS1 proteins by Hsp90 and plays an important role in the precise regulation of cell migration. Depletion of NudCL2 leads to myosin-9 and LIS1 degradation and increases single-cell migration but not collective cell migration.

Given that NudCL2 not only functions as an Hsp90 cochaperone, but also enhances myosin-9 stability, we attempted to assess whether Hsp90 is involved in myosin-9 stability and cell migration in mammalian cells. Immunoprecipitation with anti-myosin-9 antibody revealed that myosin-9 was able to interact with Hsp90 (Fig. 7a). Inhibition of Hsp90 ATPase activity by geldanamycin (GA) not only decreased myosin-9 protein levels, impaired actin stress fiber and focal adhesion formation, but also led to increase in single-cell migration (Fig. 7b–h). Similar results were also observed in A549 cells treated with another inhibitor of Hsp90, radicicol (RA) (Supplementary Fig. 5). Together, these data indicate that Hsp90 plays an important role in myosin-9 protein stability and cell migration regulation.

Since either depletion of NudCL2 or inhibition of Hsp90 ATPase activity destabilizes myosin-9 and promotes single-cell motility (Figs. 1, 2, 7a–h), we speculated that Hsp90 is involved in the regulation of myosin-9 by NudCL2. We designed a series of rescue experiments and found that the exogenous expression of Hsp90 efficiently reversed myosin-9 downregulation and defects in actin organization and focal adhesion formation induced by NudCL2 depletion (Fig. 7i–l). In contrast, ectopic expression of NudCL2 failed to reverse the phenotypes caused by Hsp90 inhibition, including myosin-9 protein instability and defects in actin organization and focal adhesion formation (Fig. 7m–p). Furthermore, depletion of NudCL2 had no synergistic effect with Hsp90 inhibition on the protein stability of myosin-9 and the organization of actin cytoskeleton (Fig. 7q–t). Taken together, these data strongly suggest that Hsp90 participates in the regulation of myosin-9 by NudCL2.

## Discussion

Cell migration, including single and collective cell migration, plays pivotal roles in many biological processes^5,25,26^; however, its underlying mechanism still remains unclear. In our study, we find that either knockdown of NudCL2 or knockout of NudCL2 significantly increased single-cell migration, but not collective cell migration in mammalian cells (Fig. 1, Supplementary Figs. 1–3). Ectopic expression of NudCL2 efficiently reverses the increase in single-cell migration induced by NudCL2 depletion (Fig. 1, Supplementary Figs. 1–3). Further data show that NudCL2 stabilizes myosin-9 and LIS1 by Hsp90. Co-depletion of myosin-9 and LIS1 phenocopies the NudCL2 depletion (Fig. 6). Thus, these data suggest that NudCL2 precisely regulates cell migration by stabilizing both myosin-9 and LIS1 with Hsp90, providing a previously undescribed regulation mechanism for cell migration (Fig. 7u).

Despite there exist two types of cell migration, single and collective cell migration, from the embryonic development to adult homeostasis in vertebrate, few studies have distinguished the different roles of them in vivo^4,5^. Accumulating studies indicate that both single and collective cell migration are involved in development and cancer metastasis^27–31^. Single-cell migration, such as rapid leukocyte migrate, plays important roles during immune response^32,33^. In contrast, collective cell migration has a key role during wound healing^34,35^. Here, we show, for the first time, that downregulation of NudCL2 significantly increases single-cell migration, but has no effect on collective cell migration, which is able to reverse by ectopic expression of NudCL2 (Fig. 1, Supplementary Figs. 1–3), suggesting that NudCL2 is essential for single-cell migration in vitro. To our best knowledge, these findings suggest that NudCL2 may be has an important role in single-cell migration-related biological process, which will be explored more thoroughly in the future.

Myosin-9 plays an important role in cell migration by regulating actin dynamics^9,10,14^. Previous studies have shown that the phosphorylation of myosin-9 by PKC and CK II is important for NM IIA dynamics^11,36^. However, the regulating mechanism of myosin-9 protein stability remains unclear. Here, we find that myosin-9 interacts with NudCL2 (Fig. 2). Depletion of NudCL2 induces the degradation of myosin-9 via the ubiquitin-proteasome pathway (Fig. 2). Further data also show that that myosin-9 interacts with Hsp90. Inhibition of Hsp90 ATPase activity destabilizes myosin-9 (Fig. 7). Exogenous expression of Hsp90 reverses myosin-9 degradation in NudCL2-depleted cells (Fig. 7). Importantly, the ectopic expression of myosin-9 is able to reverse the increase in cell migration induced by NudCL2 depletion (Fig. 4). Taken together, these data indicate that myosin-9 is stabilized by NudCL2 through the Hsp90 pathway.

The cytoskeleton, including actin and microtubules, spatially and temporally regulates cell migration^37,38^. Growing evidence has indicated that there are crosstalks between actin and microtubule during cell migration^38–40^. Rho-family GTPases, such as Ras homolog A (RhoA), Rac family small GTPase 1 (Rac1) and cell division cycle 42 (Cdc42), are important players in mediating the dynamics of both actin and microtubule^38,40^. Furthermore, myosin IIA is involved in cell motility regulation and actin-microtubule crosstalk by regulating Rac1 activity^20^. Our previous works has revealed that NudCL2 depletion destabilized LIS1 and led to a marked perinuclear accumulation of microtubules^18^. In this report, we find that knockdown of NudCL2 decreases the protein levels in both myosin-9 and LIS1, two important regulators of both actin and microtubule dynamics^10,41^, and causes disorganization of cytoskeleton (Figs. 2, 3, 5, Supplementary Fig. 6a, b). NudCL2 knockdown causes the degradation of both LIS1 and myosin-9 (Fig. 5). However, depletion of myosin-9 or LIS1 exhibits the opposite effects on cell migration (Fig. 5, Supplementary Fig. 4). Interestingly, co-depletion of myosin-9 and LIS1 induces the migration phenotypes similar to those observed in NudCL2-downregulated cells (Fig. 6). Together, these data suggest that NudCL2 may act as a regulator of both actin and microtubule by stabilizing myosin-9 and LIS1 to precisely orchestrate cell migration.

Accumulating studies have shown that myosin-9 and LIS1 play an opposite role in cell migration regulation^20,22–24^. Reduction of myosin-9 promotes cell migration by impairing the structure and function of actin cytoskeleton (Supplementary Figs. 4, 6c, d)^8,14,20^. However, downregulation of LIS1 inhibits cell migration by perturbing microtubule dynamics (Fig. 5, Supplementary Fig. 6e, f)^23,24,48,49^. Here, we provide evidence that NudCL2 stabilizes both myosin-9 and LIS1, and regulates both actin and microtubule dynamics (Figs. 2, 5, Supplementary Fig. 6a, b). Together, these results suggest that NudCL2 may regulate cell migration by stabilizing both myosin-9 and LIS1 through synergistically modulating actin and microtubule dynamics.

## Materials and methods

### Plasmids and siRNAs

The human *Flag*-*NudCL2, Flag*-NudCL2* (with the silent mutation of three nucleic acids in the siRNA-targeting region: ACCTTGAAAAGTGACTGCT), *GST*-*NudCL2, Myc*-*NudCL2*, and *Myc*-*Hsp90* vectors were previously constructed by our group^15,18^. Full-length human *MYH9* cloned by RT-PCR from A549 cells was inserted into pEGFP-N1 (Clontech, Palo Alto, CA, USA). All of these constructs were confirmed by DNA sequencing.

All siRNAs were synthesized by GenePharma (Shanghai, China). The sequences of the sense strands of the siRNA duplexes were as follows:

*LIS1:* 5′-CGGACAAGTAGAATAAATG-3′^18^

*MYH9-1:* 5′-GAUCAAUCCAUCUUGUGCATT-3′^22^

*MYH9-2:* 5′-UCUUGUGCUACUCUAGGATT-3′^22^

*NudCL2-1:* 5′-ACCUUGAGAAAUAACTGCUTT-3′^15^

*NudCL2-2:* 5′-CAAGGGCAAACUCUUUGAUTT-3′^15^.

### Generation of NudCL2 knockout cell lines with CRISPR/Cas9-mediated genome editing

CRISPR/Cas9 plasmid targeting the first exon of the *NudCL2* gene was constructed previously^19^. NudCL2 knockout A549 cell lines were generated as described previously^19^. In brief, A549 cells were transfected with CRISPR/Cas9 plasmid for 48 h, followed by treatment with 1 µg/ml puromycin for 48 h. After selection, the cells were counted, diluted to a density of 1 cell per 200 μl of medium and seeded into 96-well plates to obtain single colonies. Western blotting and genomic DNA sequencing were used to identify NudCL2 knockout colonies. The primers used to amplify the target region were as follows^19^:

Forward: 5′-AGGCGTAGCCTAAGCGTGGGATTC-3′

Reverse: 5′-ACCCAACAGTCGTTCAGGGAAACG-3′.

### Cell culture and transfections

A549 cells (preserved in our laboratory) were maintained in Roswell Park Memorial Institute 1640 (RPMI 1640, Corning, Shanghai, China) medium supplemented with 10% fetal bovine serum (FBS, PAA Laboratories, Northbrook, IL, USA) at 37 °C in 5% CO_2_. HeLa and HEK-293 cells (preserved in our laboratory) were maintained in Dulbecco’s modified Eagle’s medium (DMEM, Corning) with 10% serum at 37 °C in 5% CO_2_. A549, HeLa and HEK-293 cell lines were tested for mycoplasma contamination before using for functional analysis. Plasmid transfection was carried out using PolyJet (SignaGen Laboratories, Rockville, MD, USA), and the siRNA duplexes were transfected with Lipofectamine RNAiMAX (Invitrogen, Carlsbad, CA, USA). The transfection process was performed according to the manufacturer’s instructions.

### Drug treatments

GA (Tocris, Missouri, UK) and RA (Tocris) were stored at −20 °C as stock solutions at 1.78 mM in DMSO and ethanol, respectively. Cells were treated with GA or RA for the indicated times as described in the text. The final concentration of GA and RA used to treat A549 cells was 3.78 µM. MG132 (Millipore, Billerica, MA, USA) was stored at −20 °C as a stock solution at 5 mM in DMSO. MG132 (10 μM) was added to A549 cells for 4 h.

### Antibodies

For immunofluorescence, antibody against paxillin (612405) (BD Biosciences, Two Oak Park, Bedford, MA, USA) was used. For western blotting analysis, antibodies against β-actin (T1978) (Sigma-Aldrich, St. Louis, MO, USA), ACTN1 (db1866) (Diagbio, Hangzhou, China), ACTN4 (19096-1-AP), FLNA (67133-1-Ig), FLNB (20685-1-AP), Hsp90 (13171-1-AP), Myosin-9 (11128-1-AP) (Proteintech, Wuhan, China), LIS1 (a12643), LRPPRC (A3365), SPTBN1 (A5253) (Abclonal, Wuhan, China), PLEC (BS91090) (Biogot, Najing, China), GFP (sc-9996) (Santa Cruz, Dallas, Texas, USA), PRMT5 (YN3030), and SPTAN1 (YC0074) (Immunoway, Plano, Texas, USA) were utilized. Anti-NudCL2 antibody was generated as described previously^18^. The secondary antibodies used for immunofluorescence analyses were Alexa Fluor 488- and 568-conjugated anti-rabbit or anti-mouse IgG (Invitrogen). Goat anti-mouse or anti-rabbit secondary antibody (LI-COR, Lincoln, NE, USA) conjugated to either Alexa Fluor 680 or IRDye 800 was used for western blotting analysis.

### Immunofluorescence staining

A549 cells grown on glass coverslips were fixed for 15 min with 4% paraformaldehyde at room temperature and then incubated with primary antibodies for 2 h and secondary antibodies for 1 h at room temperature. Phalloidin (SignaGen, P1951) was used to stain actin. DNA was stained with DAPI (Sigma). The mounted coverslips were analyzed by confocal fluorescence microscopy with an oil immersion ×63 objective (Zeiss, LSM880, USA).

### GST pull-down assays

GST pull-down assays were performed as described previously^42^. GST and GST-NudCL2 were purified from bacteria. To detect the association between NudCL2 and myosin-9, blots were probed with antibodies as indicated in the text.

### Immunoprecipitation and western blot assays

Immunoprecipitation for endogenous proteins was performed as described previously^42^. Briefly, whole-cell extracts were generated in TBSN buffer (20 mM Tris [pH 8.0], 150 mM NaCl, 0.5% Nonidet P-40, 5 mM EGTA, 1.5 mM EDTA, 0.5 mM Na_3_VO_4_, 20 mM p-nitrophenyl phosphate) supplemented with protease inhibitors and subjected to coimmunoprecipitation analysis with the indicated antibodies. Western blotting analyses were performed with the indicated antibodies and analyzed using the LI-COR Odyssey (LI-COR) system.

### Quantitative real-time RT-PCR

Quantitative RT-PCR analyses for *MYH9* were performed using a Bio-Rad CFX-Touch System with HiScript Q RT SuperMix (Vazyme, Nanjing, China). All of the reactions were performed in triplicate. GAPDH served as an internal control. The primers used to amplify the target regions were as follows:

Forward: 5′-ATCTCGTGCTATCCGCCAAG-3′

Reverse: 5′-GTTGTACGGCTCCAACAGGA-3′.

### Cell tracking

Cells were transfected with the indicated siRNAs and vectors for 72 h. Then, time-lapse video microscopy was used to track cell migration. Fluorescence images were captured at 5-s intervals for 500 cycles with an LSM880 confocal microscope (Zeiss). The videos were further analyzed using Imaris 9.1.2 software.

### Kymography analysis

For kymography, phase-contrast time-lapse sequences were captured using a ×63 oil immersion objective on an LSM880 Zeiss confocal microscope. Movies were recorded for 10–15 min at a rate of one frame per 3 s. Kymographs were produced and analyzed by MetaMorph software (Molecular Devices, Sunnyvale, CA, USA). Kymographs were generated along 1-pixel-wide line regions oriented in the direction of individual protrusions. Quantitative analysis of kymographs was performed as previously described^43^.

### Transwell assay

The migratory potential was measured using a Transwell chamber (8-µm pore, Corning). Two hundred microliters of a suspension containing 80,000 transfected cells was plated in medium with 1% FBS in the top chamber of a transwell apparatus, while seven hundred microliters of medium containing 20% FBS was placed in the lower well. After 24 h of incubation, cells were fixed in 4% paraformaldehyde for 15 min and dyed with 0.1% crystal violet for 20 min. Cells in five fields in each well were captured under a microscope (magnification ×200).

### Wound healing assay

Scratch wound assays were performed as described previously^44^. Briefly, transfected cells were reseeded into 30 mm dishes with 10% serum-containing culture medium. When the cells became confluent, the cells were starved for 12 h and scratched with a 20 μl pipette tip to create wounds. The cells were washed to remove debris and then replaced with 1% serum culture medium to allow wound healing. The cells were monitored with a microscope, and representative images were taken at the indicated time points. The pictures were quantified by using ImageJ software.

### LC-MS/MS analysis and database searching

The LC-MS/MS analysis and database searching were performed as described previously^45^. Briefly, HeLa cells transfected with the Flag-NudCL2 vector were subjected to immunoprecipitation purification with anti-FLAG antibody-coupled beads (Sigma). The proteins were subjected to trypsin digestion, and the recovered peptide mixtures were separated by reversed-phase HPLC followed by tandem mass analysis by the Reach Center for Proteome Analysis, Shanghai Institutes of Biological Sciences (Shanghai, China)^46^. The peak lists of all acquired MS/MS spectra were generated by BioWorks software and then automatically searched against the human International Protein Index protein sequence database (version 3.36) with the SEQUEST algorithm^47^.

### Statistical analysis

In order to choose the sample size, statistical analysis was performed according to previous reports^15,20^. The actual sample size with sufficient statistical power for each experiment is provided in the figures. The cells for analysis were from random fields. Data are representative of at least three independent experiments. Means and standard deviations (SD) were calculated for all quantitative experiments. Two-tailed Student’s *t-*test was used to determine statistically significant differences between two groups (GraphPad Prism 6). Statistical significance was specified as *P < 0.05, **P < 0.01, or ***P < 0.001.

## Supplementary information

Supplementary figure 1

Supplementary figure 2

Supplementary figure 3

Supplementary figure 4

Supplementary figure 5

Supplementary figure 6

Supplementary Table 1

Supplementary information
