# Correction: NudCL2 regulates cell migration by stabilizing both myosin-9 and LIS1 with Hsp90

**DOI:** 10.1038/s41419-024-07256-7

**Published:** 2024-12-10

**Authors:** Wenwen Chen, Wei Wang, Xiaoxia Sun, Shanshan Xie, Xiaoyang Xu, Min Liu, Chunxia Yang, Min Li, Wen Zhang, Wei Liu, Liangjing Wang, Tianhua Zhou, Yuehong Yang

**Affiliations:** 1 Department of Cell Biology, and Institute of Gastroenterology of the Second Affiliated Hospital, Zhejiang University School of Medicine, Hangzhou, Zhejiang China; 2 Shanghai Key Laboratory of Psychotic Disorders, Shanghai Mental Health Center, Shanghai Jiao Tong University School of Medicine, Shanghai, China; 3 The Cancer Center of the Second Affiliated Hospital, Zhejiang University School of Medicine, Hangzhou, Zhejiang China; 4 Collaborative Innovation Center for Diagnosis and Treatment of Infectious Diseases, Hangzhou, Zhejiang China; 5 Department of Molecular Genetics, University of Toronto, Toronto, ON Canada

Correction to: *Cell Death and Disease* 10.1038/s41419-020-02739-9, published online 14 July 2020

The original version of this article unfortunately contained errors in Fig. 1j. We have carefully checked our original data and corrected Fig. 1j. We apologize for the errors and state that this correction does not affect either the results or the conclusions of the paper.
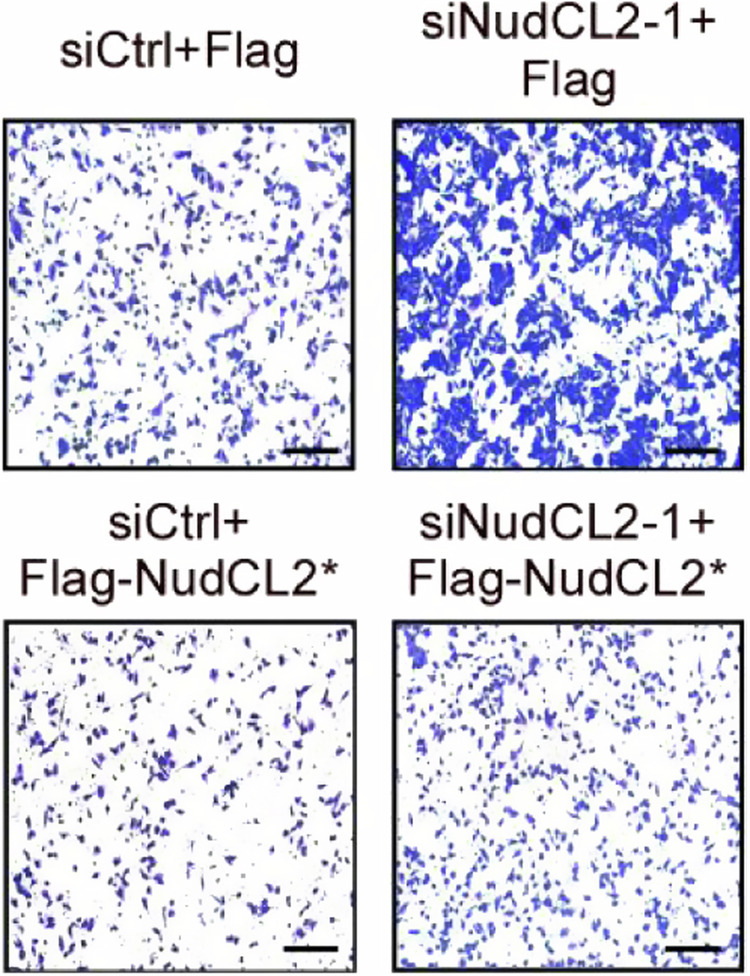


Uncorrected figure
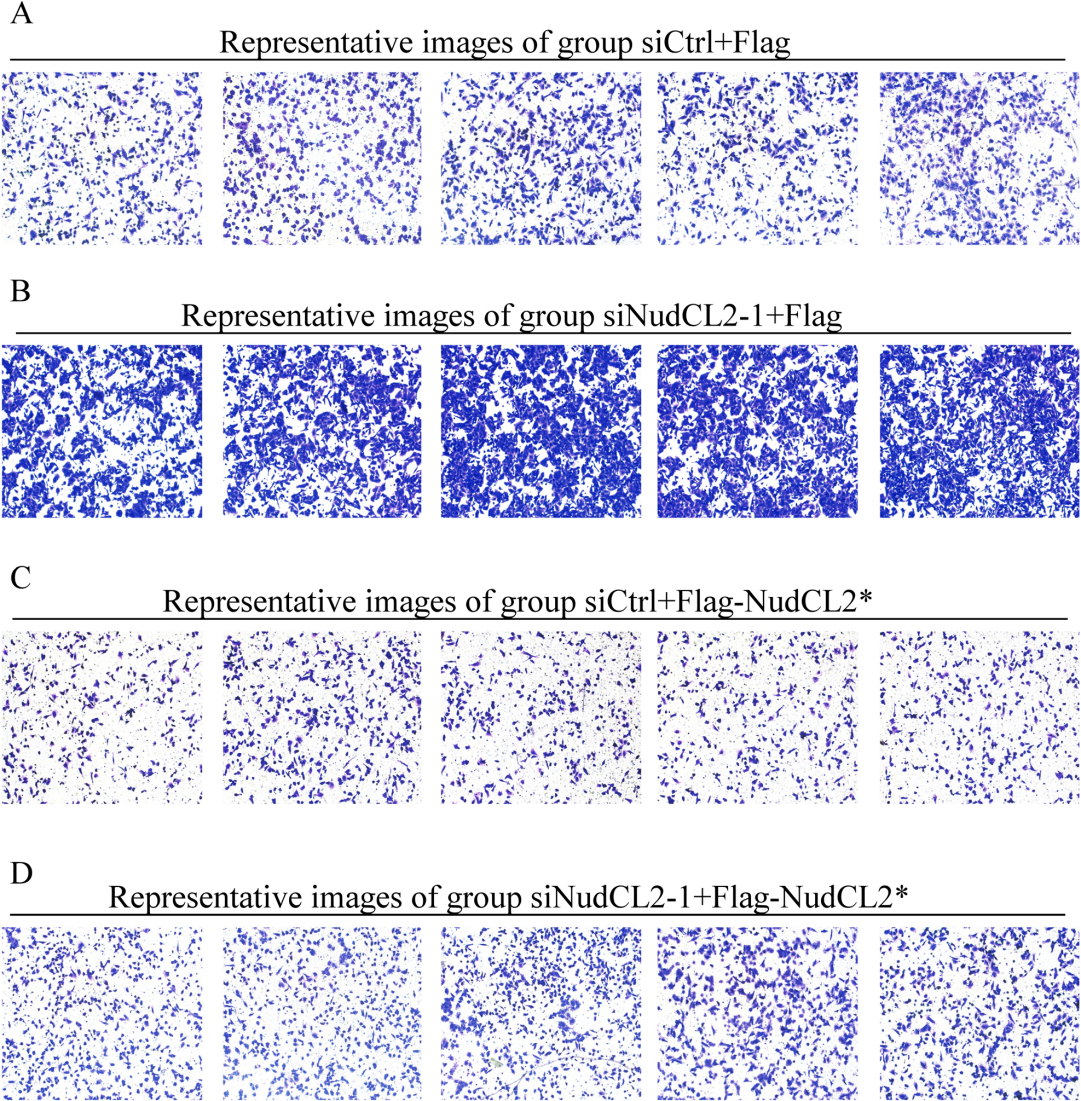


The original article has been corrected.

## Supplementary information

Original data

