# Supplementary figures and images for: NudCL2 regulates cell migration by stabilizing both myosin-9 and LIS1 with Hsp90
Source: Cell Death Dis. 2020 Jul 14;11(7):534. doi: 10.1038/s41419-020-02739-9 (PMC7360774; doi:10.1038/s41419-020-02739-9)

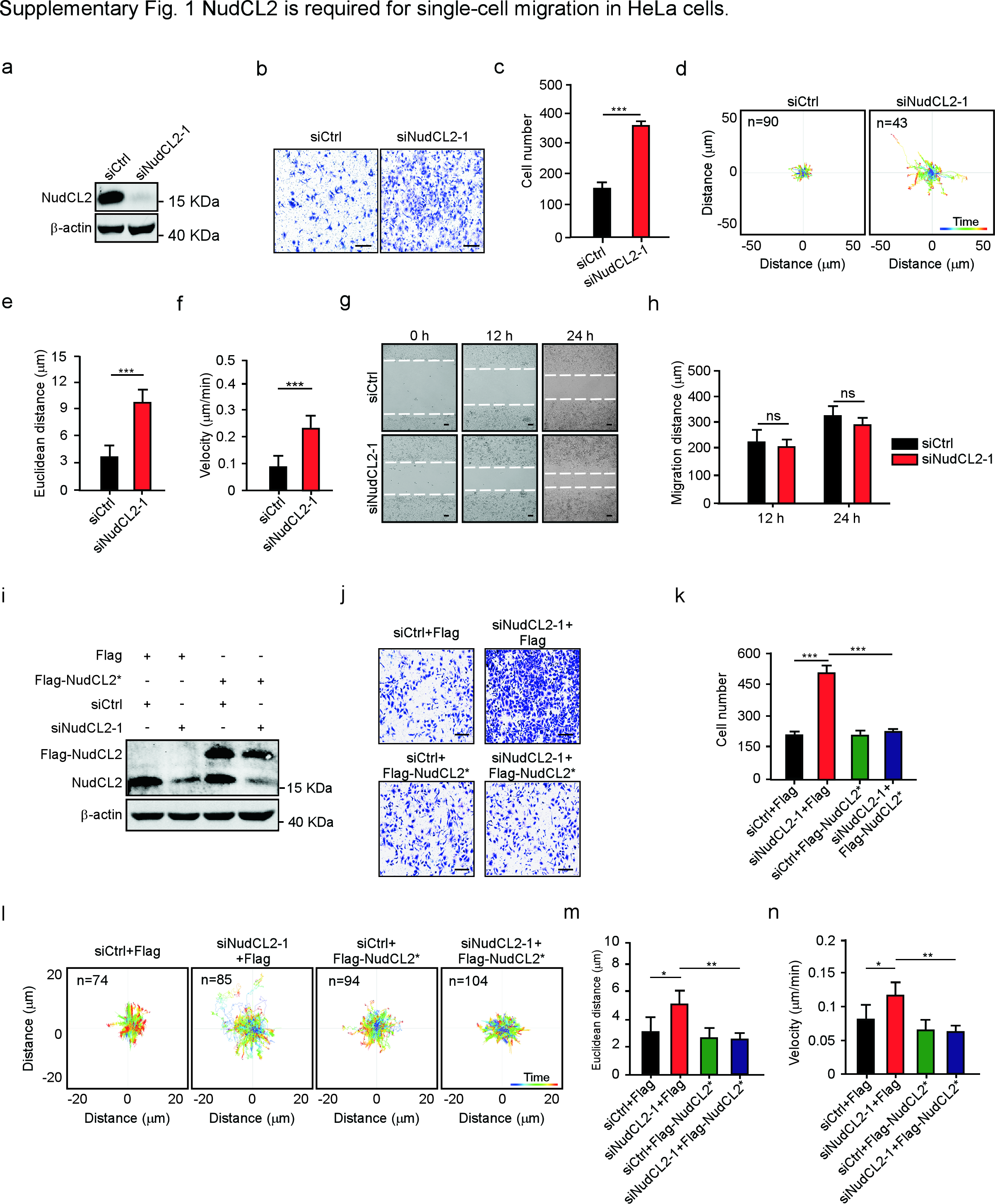

Supplement: Supplementary file 1 — Supplementary figure 1 [file 41419_2020_2739_MOESM1_ESM.tif]

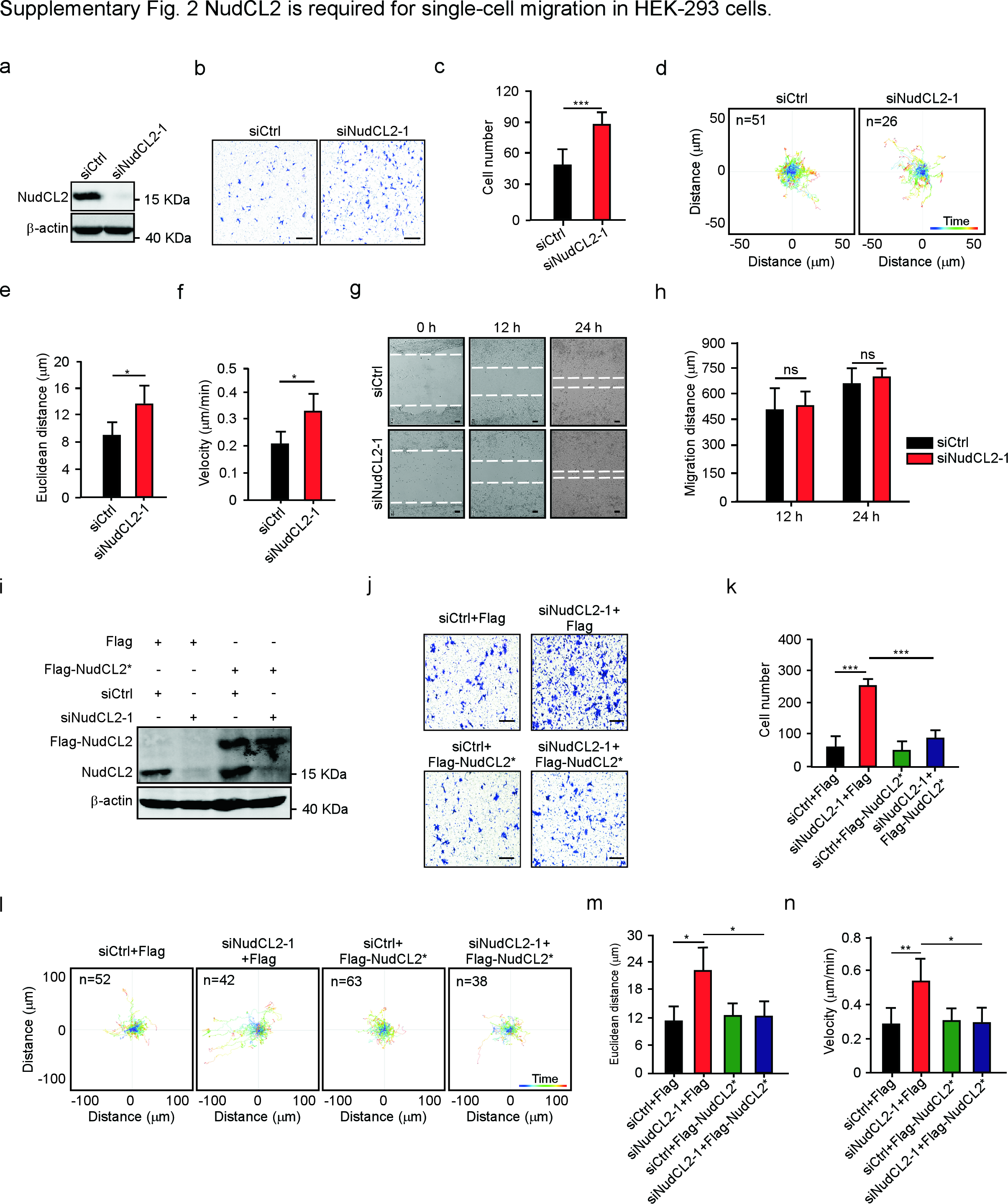

Supplement: Supplementary file 2 — Supplementary figure 2 [file 41419_2020_2739_MOESM2_ESM.tif]

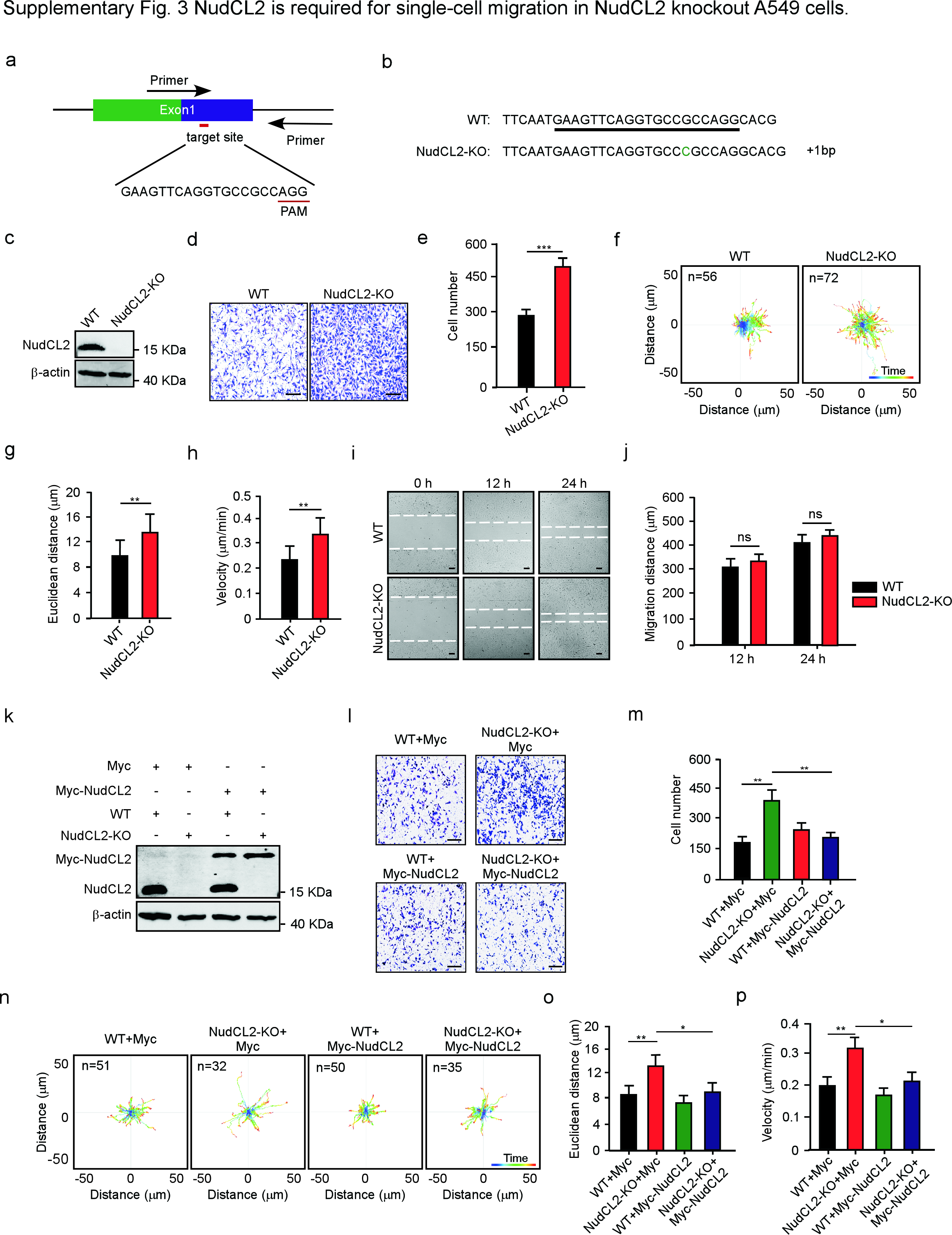

Supplement: Supplementary file 3 — Supplementary figure 3 [file 41419_2020_2739_MOESM3_ESM.tif]

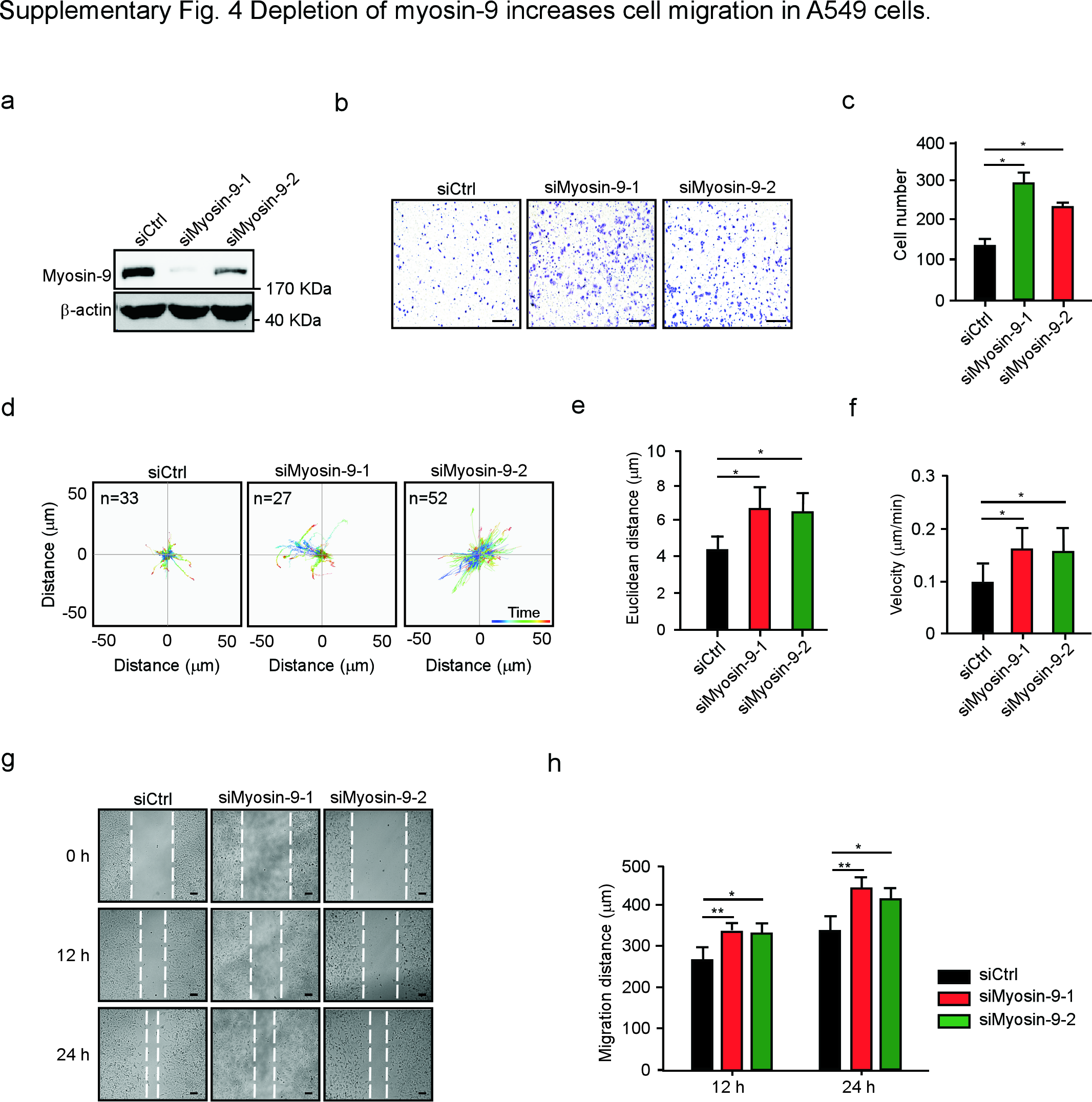

Supplement: Supplementary file 4 — Supplementary figure 4 [file 41419_2020_2739_MOESM4_ESM.tif]

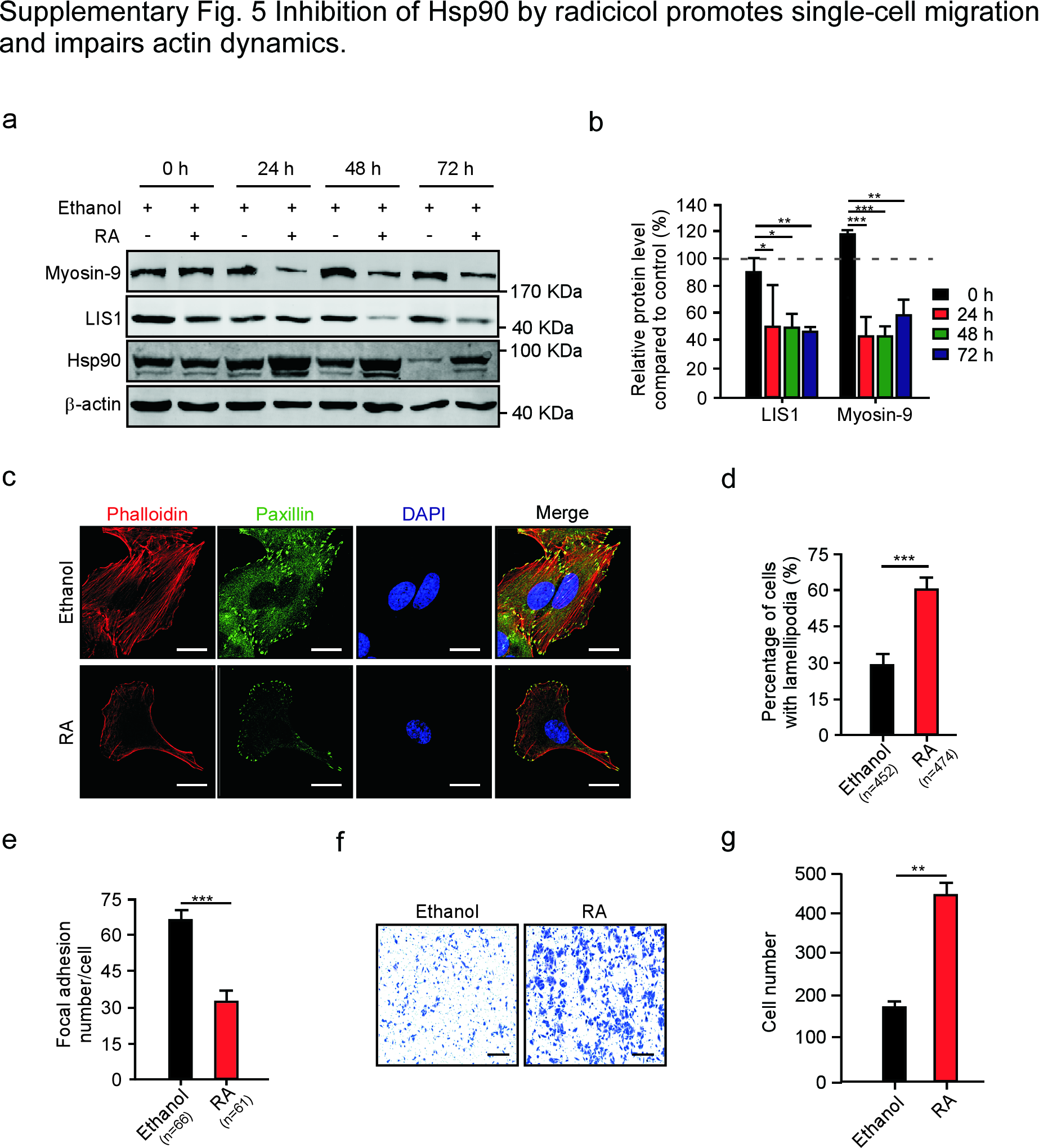

Supplement: Supplementary file 5 — Supplementary figure 5 [file 41419_2020_2739_MOESM5_ESM.tif]

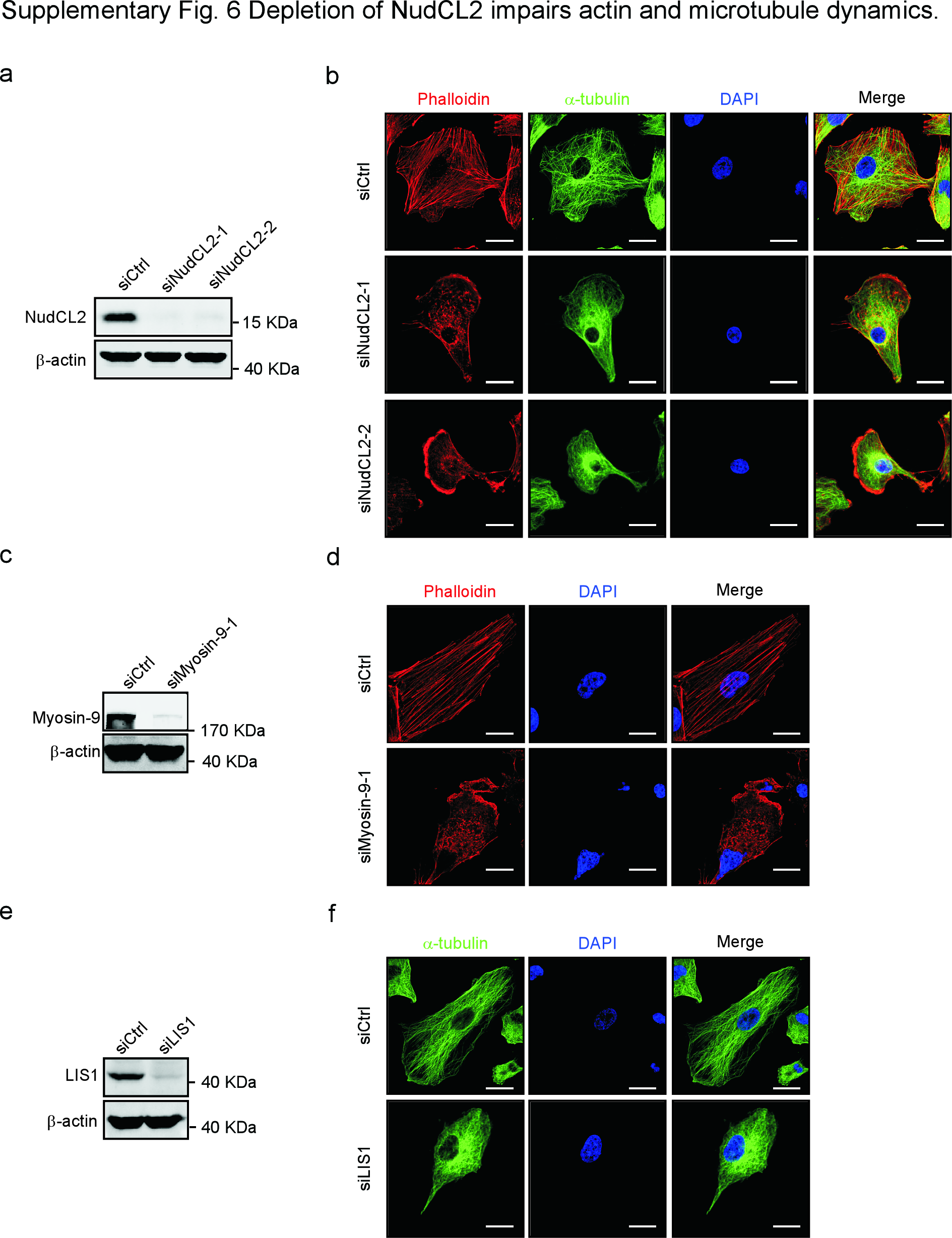

Supplement: Supplementary file 6 — Supplementary figure 6 [file 41419_2020_2739_MOESM6_ESM.tif]
