# Supplementary material for: NudCL2 regulates cell migration by stabilizing both myosin-9 and LIS1 with Hsp90
Source: Cell Death Dis. 2020 Jul 14;11(7):534. doi: 10.1038/s41419-020-02739-9 (PMC7360774; doi:10.1038/s41419-020-02739-9)
Supplement: Supplementary file 8 — Supplementary information [file 41419_2020_2739_MOESM8_ESM.doc]

**Supplemental figure legends**

**Supplementary Fig. 1 NudCL2 is required for single-cell migration in HeLa cells**. HeLa cells transfected with siRNA were subjected to the following analyses: **a** Western blotting analysis of the expression of NudCL2. β-actin, a loading control. **b, c** Transwell migration assays showed the cell motility of control and NudCL2-depleted cells. Scale bar, 200 μm. Cells that migrated to the undersides of the filters were counted. **d-f** The migration tracks of individual cells transfected with the indicated siRNAs were traced by Imaris 9.1.2 software. Representative single-cell migration paths are shown. Euclidean distance and migration velocity were calculated. **g, h** The wound healing assays showed collective cell migration at different time points. Dashed lines indicate the wound edges. Scale bar, 200 μm. The distance of the wound was measured by ImageJ software. **i-k** Cells transfected with the indicated siRNAs and Flag-NudCL2* (siRNA-resistant NudCL2) or Flag were subjected to the following analyses. Western blotting analysis revealed the expression of NudCL2 and Flag-NudCL2. β-actin, a loading control. Transwell migration assays revealed cell motility. Scale bar, 200 μm. Cells that migrated to the undersides of the filters were counted. **l-n** Cells transfected with the indicated siRNAs and vectors for 72 h were subjected to a migration experiment. The migration paths of the individual cells were analyzed with Imaris 9.1.2 software. Representative single-cell migration tracks are shown. Euclidean distance and migration velocity were measured. Quantitative data from at least three independent experiments are shown as the mean ± SD. n, sample size. * *P* < 0.05; ** *P* < 0.01; *** *P* < 0.001; ns, not significant (*P* > 0.05). Student’s *t*-test.

**Supplementary Fig. 2 NudCL2 is required for single-cell migration in HEK-293 cells**. HEK-293 cells transfected with siRNA were subjected to the following analyses: **a** Western blotting analysis of the expression of NudCL2. β-actin was used as a loading control. **b, c** Transwell migration assays revealed the cell motility of control and NudCL2-depleted cells. Scale bar, 200 μm. Cells that migrated to the undersides of the filters were counted. **d-f** The migration tracks of individual cells transfected with the indicated siRNAs were traced by Imaris 9.1.2 software. Representative single-cell migration paths are shown. Euclidean distance and migration velocity were calculated. **g, h** The wound healing assays displayed collective cell migration at different time points. Dashed lines indicate the wound edges. Scale bar, 200 μm. The distance of the wound was measured by ImageJ software. **i-k** Cells transfected with the indicated siRNAs and Flag-NudCL2* (siRNA-resistant NudCL2) or Flag were subjected to the following analyses. Western blotting analysis showed the expression of NudCL2 and Flag-NudCL2. β-actin was served as a loading control. Transwell migration assays revealed cell motility. Scale bar, 200 μm. Cells that migrated to the undersides of the filters were counted. **l-n** Cells transfected with the indicated siRNAs and vectors for 72 h were subjected to a migration experiment. The migration paths of the individual cells were analyzed with Imaris 9.1.2 software. Representative single-cell migration tracks are shown. Euclidean distance and migration velocity were measured. Quantitative data from at least three independent experiments are shown as the mean ± SD. n, sample size. * *P* < 0.05; ** *P* < 0.01; *** *P* < 0.001; ns, not significant (*P* > 0.05). Student’s *t*-test.

**Supplementary Fig. 3 NudCL2 is required for single-cell migration in NudCL2 knockout A549 cells.** **a** Knockout of NudCL2 in A549 cells via the CRISPR/Cas9 system. Schematic diagram of sgRNA designed for NudCL2 knockout. **b** Mutated inserts containing the NudCL2 DNA locus in NudCL2 knockout cell lines. **c** Western blotting analysis of NudCL2 expression in wild-type (WT) and NudCL2-KO cells. β-actin was used as a loading control. **d, e** Transwell migration assays revealed the cell motility of the WT and NudCL2-KO cells. Scale bar, 200 μm. Cells that migrated to the undersides of the filters were counted. **f-h** The migration tracks of individual WT and NudCL2-KO cells were traced by Imaris 9.1.2 software. Representative single-cell migration paths are shown. Euclidean distance and migration velocity were calculated. **i, j** The wound healing assays showed collective cell migration at different time points. Dashed lines indicate the wound edges. Scale bar, 200 μm. The distance of the wound was measured by ImageJ software. **k-m** Cells transfected with the Myc-NudCL2 or Myc were subjected to the following analyses. Western blotting analysis showed the expression of NudCL2 and Myc-NudCL2. β-actin was used as a loading control. Transwell migration assays revealed cell motility. Scale bar, 200 μm. Cells that migrated to the undersides of the filters were counted. **n-p** Cells transfected with the indicated vectors for 72 h were subjected to a migration experiment. The migration paths of the individual cells were analyzed with Imaris 9.1.2 software. Representative single-cell migration tracks are shown. Euclidean distance and migration velocity were measured. Quantitative data from at least three independent experiments are shown as the mean ± SD. n, sample size. * *P* < 0.05; ** *P* < 0.01; *** *P* < 0.001; ns, not significant (*P* > 0.05). Student’s *t*-test.

**Supplementary Fig. 4 Depletion of myosin-9 increases cell migration in A549 cells.** Cells transfected with siRNAs were subjected to the following analyses: **a** Western blotting analysis of the expression of myosin-9. β-actin was used as a loading control. **b, c** Transwell migration assays revealed the cell motility of control and myosin-9-depleted cells. Scale bar, 200 μm. Cells that migrated to the undersides of the filters were counted. **d-f** The migration tracks of individual cells were traced by Imaris 9.1.2 software. Representative single-cell migration paths are shown. Euclidean distance and migration velocity were measured. **g, h** Wound healing assays showed collective cell migration at different time points. Dashed lines indicate wound edges. Scale bar, 200 μm. The distance of the wound was measured by ImageJ software. Quantitative data derived from at least three independent experiments are shown as the mean ± SD. n, sample size. * *P* < 0.05; ** *P* < 0.01. Student’s *t*-test*.*

**Supplementary Fig. 5 Inhibition of Hsp90 by radicicol promotes single-cell migration and impairs actin dynamics.** A549 cells treated with radicicol (RA) or ethanol for 48 h were used for the following analyses: **a** Western blotting analysis of the expression of myosin-9, LIS1 and Hsp90. β-actin was used as a loading control. **b** Relative protein levels compared to the control at the same time point of RA treatment in Supplementary Fig. 5a were measured using Image J software and shown. **c-e** Cells were fixed and stained with phalloidin (red) and anti-paxillin (green) antibody. DNA was visualized with DAPI (blue). Scale bar, 20 μm. The percentage of cells with lamellipodia was calculated, and cellular focal adhesions were counted. **f, g** Transwell migration assays revealed the cell motility of cells treated with RA or ethanol for 48 h. Scale bar, 200 μm. Cells that migrated to the undersides of the filters were counted. Quantitative data derived from at least three independent experiments are shown as the mean ± SD. More than 150 cells were counted in each experiment. n, sample size. * *P* < 0.05; ** *P* < 0.01; *** *P* < 0.001. Student’s *t*-test.

**Supplementary Fig. 6 Depletion of NudCL2 impairs actin and microtubule dynamics. a, b** A549 cells transfected with control or NudCL2 siRNA were subjected to western blotting analysis and immunofluorescence with the indicated antibodies. β-actin, a loading control. Actin was stained with phalloidin (red). Microtubule was stained with anti-α-tubulin antibody (green). DNA was visualized with DAPI (blue). Scale bar, 20 μm. **c, d** A549 cells transfected with control or myosin-9 siRNA were subjected to western blotting analysis with anti-myosin-9 antibody and immunofluorescence with phalloidin (red). β-actin, a loading control. DNA was visualized with DAPI (blue). Scale bar, 20 μm. **e, f** A549 cells transfected with control or LIS1 siRNA were subjected to western blotting analysis with LIS1 antibody and immunofluorescence with anti-α-tubulin antibody (green). β-actin, a loading control. DNA was visualized with DAPI (blue). Scale bar, 20 μm.

**Supplementary Table 1. NudCL2-interacting proteins characterized by IP/MS (The proteins are ranked by relative abundance)**
